# Biosynthesis, Characterization, and Antibacterial Activity of Gold, Silver, and Bimetallic Nanoparticles Using *Annona squamosa* L. Leaves

**DOI:** 10.3390/antibiotics13121199

**Published:** 2024-12-09

**Authors:** Fatima Jibrin, Olufunto T. Fanoro, Rodney Maluleke, Thabang C. Lebepe, Nande Mgedle, Gracia It Mwad Mbaz, Olanrewaju A. Aladesuyi, Rajendran Kalimuthu, Oluwatoyin A. Odeku, Oluwatobi S. Oluwafemi

**Affiliations:** 1 Pan African University Life and Earth Sciences Institute (PAULESI), Ibadan 200132, Nigeria; missfatimajibrin@gmail.com; 2 Department of Pharmacognosy, University of Ibadan, Ibadan 200132, Nigeria; 3 Department of Chemical Sciences (Formerly Applied Chemistry), University of Johannesburg, Doornfontein, Johannesburg 2028, South Africa; jolufunto@gmail.com (O.T.F.); rodney.maluleke@gmail.com (R.M.); calvyn.tl@gmail.com (T.C.L.); nandemgedle@gmail.com (N.M.); gmbazitmwad@gmail.com (G.I.M.M.); lanrefavoured@gmail.com (O.A.A.); drrajendran5050@gmail.com (R.K.); 4 Centre for Nanomaterials Sciences Research, University of Johannesburg, Doornfontein, Johannesburg 2028, South Africa; 5 Department of Pharmaceutics and Industrial Pharmacy, University of Ibadan, Ibadan 200132, Nigeria; pejuodeku@yahoo.com

**Keywords:** *Annona squamosa* L., antibacterial, *Staphylococcus aureus*, *Escherichia coli*, silver nanoparticles, gold nanoparticles, bimetallic nanoparticles

## Abstract

The utilization of nano-sized drug delivery systems in herbal drug delivery systems has a promising future for improving drug effectiveness and overcoming issues connected with herbal medicine. As a consequence, the use of nanocarriers as novel drug delivery systems for the improvement of traditional medicine is critical to combating infectious diseases globally. In line with this, we herein report the biosynthesis of silver nanoparticles (AgNPs), gold nanoparticles (AuNPs), and bimetallic nanoparticles (BMNPs) as antibacterial agents against pathogenic bacterial strains using *Annona squamosa* L. leaf extract as a bio-reductant and bio-stabilizing agent. The as-synthesized metal nanoparticles were characterized by transmission electron microscopy (TEM), ultraviolet–visible (UV–Vis) absorption spectroscopy, X-ray diffraction (XRD), Fourier-transform infrared spectroscopy (FTIR), and the dynamic light scattering (DLS) method. The as-synthesized MNPs had an average particle size of 6.98 nm ± 2.86 nm (AgNPs), 21.84 ± 8.72 nm (AuNPs), and 2.05 nm ± 0.76 nm (BMNPs). The as-synthesized AgNPs and BMNPs showed good antibacterial activity against pathogenic bacterial strains of Gram-positive *Staphylococcus aureus* (ATCC 25923) and Gram-negative *Escherichia coli* (ATCC 25922). The obtained results offer insight into the development of benign nanoparticles as safe antibacterial agents for antibiotic therapy using *Annona squamosa* L. leaf extract.

## 1. Introduction

Medicines have been known to play a significant role in the improvement of the quality of life by reducing the rate of morbidity and mortality [1]. Metals have been utilized for centuries in several nations as medicine and antimicrobial agents. Since 1500 BCE, Egyptians have used copper salt as a sterilizing agent in wound care. Silver (Ag) and copper (Cu) have been employed by the Greeks, Persians, Romans, Indians, and Egyptians in the purification of water and the preservation of food [2]. In recent years, scientists have progressed into nanotechnology research, particularly in the synthesis, characterization, and applications of metal nanoparticles (MNPs). MNPs are specifically noble metal nanoparticles such as platinum (Pt), palladium (Pd), copper (Cu), silver (Ag), and gold (Au) nanoparticles [3]. These nanomaterials possess distinctive physical and chemical characteristics compared to bulk-sized materials, such as a relatively large surface area, which promotes chemical reactivity. In addition, optical, electrical, and magnetic properties have made them gain traction in scientific study and development [4,5]. The bactericidal properties of MNPs are largely due to their large surface area, capping agent, charge, and small size [6,7]. 

The production of biofilms and the development of multidrug-resistant (MDR) bacteria, both of which are induced by the misuse of antibiotics, are the two greatest hurdles in treating infections caused by bacteria [8]. Research on the use of metallic nanoparticles as antibiotic replacements is gaining popularity due to their potential in treating pathogenic bacterial infections. Metallic and metallic oxide-based nanoparticles possess unique properties that can offer long-term antibacterial and biofilm prevention and help distinguish mammalian cells from bacterial ones [9,10]. These nanoparticles possess antibacterial potentials because they produce reactive oxygen species (ROS) that attack prokaryotic cell structures, DNA synthesis, and metabolic processes that culminate in cell death [11,12]. Silver nanoparticles were reported to exhibit a significant antimicrobial activity compared to the leaf extract when tested against bacterial and fungal species [13]. Gold nanoparticles also show toxicity to bacteria at various doses [14]. Also, bimetallic Au-Ag nanoparticles (BMNPs), which combine the properties of two metals in a single nanostructured system, have been shown to possess good antibacterial properties [15]. Thus, new antibacterial treatments are continuously being developed using silver nanoparticles (AgNPs) and gold nanoparticles (AuNPs) [16,17,18].

Chemical and physical methods have historically been employed in synthesizing nanoparticles. Ion sputtering, hydrothermal synthesis, and sol–gel processes are among these methods. Chemical processes include chemical reduction, electrochemical treatments, and photochemical reduction. Bottom-up and top-down techniques for nanoparticle synthesis are notable methods, with the former involving the addition of building blocks to make nanoparticles and the latter involving the miniaturization or breaking down of bulk materials without compromising their original integrity [19,20]. However, the time involved, and the toxicity of the materials produced by the aforementioned method have made them unsuitable for clinical trials. Therefore, an ecologically friendly and non-toxic method of producing MNPs is via “green synthesis”. This method uses plants and other biological materials, unlike the chemical and physical methods, which often utilize toxic and expensive chemicals and require high temperature, pressure, and power which have negative impacts on the environment [21,22,23]. Plants are advantageous as bio-reductants in green synthesis due to their ease of access and preparation. In addition, they possess phytochemicals such as flavonoids, phenolics, ketones, and terpenes, which provide electrons that enable them to act as both reducing and stabilizing agents [24]. 

*Annona squamosa* L. (AS) is a medicinal, thin, tall tree or shrub from the *Annonaceae* family, with a height between 3 and 6 m. It has a conical or heart-shaped compound fruit with a base depression that is either shallow or deep [25]. The root of *A. squamosa* L. is used as a quick-action purgative, and the powdered leaves are often sniffed to overcome hysteria, decrease inflammation, and relieve ulcers and sores. The mature fruits of this plant are used to expedite suppuration in various cancers [26]. The biological activities of AS have been linked to the presence of diverse kinds of phytochemicals recorded in the plant, demonstrating pharmacological and biological effects such as antioxidant, antiviral, antidiabetic, antimicrobial, anti-obesity, antidiarrheal, and antitumor properties [27,28]. Previous reports have shown that many phytochemicals such as flavonoids, steroids, saponin, phenols, tannins, reducing sugar, and alkaloids, were found to be more abundant in the aqueous extract of the *A. squamosa* L. leaf [29,30,31,32]. Diterpenes (DITs), alkaloids (ALKs), cyclopeptides (CPs), annonaceous acetogenins (ACGs), and essential oils are its main components [28].

In this study, the synergistic role of two metal salts to produce smaller NPs was explored. The aqueous extract of AS was used as both reducing and capping agents for the synthesis of silver (Ag), gold (Au), and bimetallic (Ag-Au) NPs. The MNPs were adequately characterized. The antibacterial activity of the as-synthesized MNPs was evaluated against both Gram-positive and -negative bacteria. This research aims to improve the drug delivery of the *A. squamosa* plant because of its potential as an alternative medicine for bacterial infections. The research was partly inspired by the ethnobotanical use of the plant by herbal healers and traditional medicine practitioners in Bassa, Kogi State, Nigeria, for treating large recalcitrant wounds and infections.

## 2. Results and Discussion

### 2.1. UV–Visible Spectra of AgNPs, AuNPs, and BMNPs Using the Aqueous Extract of AS 

**Figure 1:**
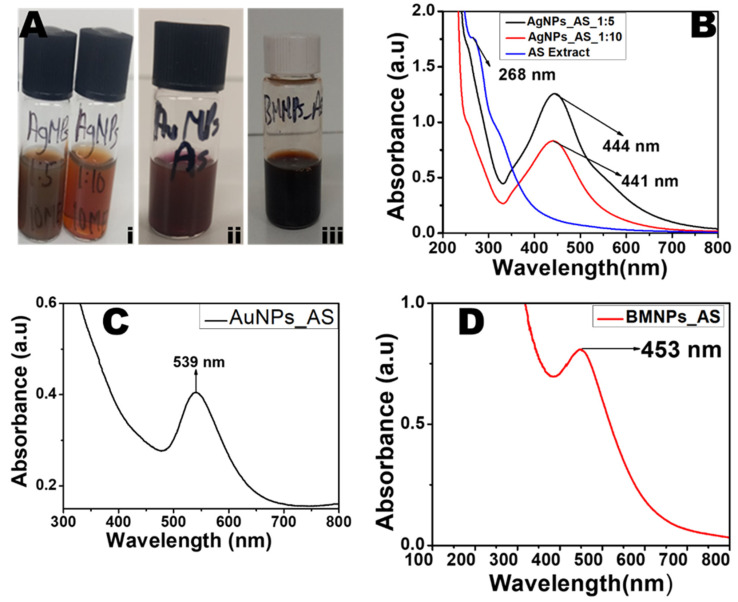
Photographic image of (**A**(**i**)) AgNPs, (**A**(**ii**)) AuNPs, and (**A**(**iii**)) BMNPs biosynthesized from *A. squamosa* L. extract. (**B**) Absorption spectra of AgNPs synthesized using the aqueous extract of AS at ratios of 1:5 and 1:10. (**C**) Absorption spectra of AuNPs synthesized using the aqueous extract of AS. (**D**) Absorbance spectra of BMNPs_AS.

**Figure 2:**
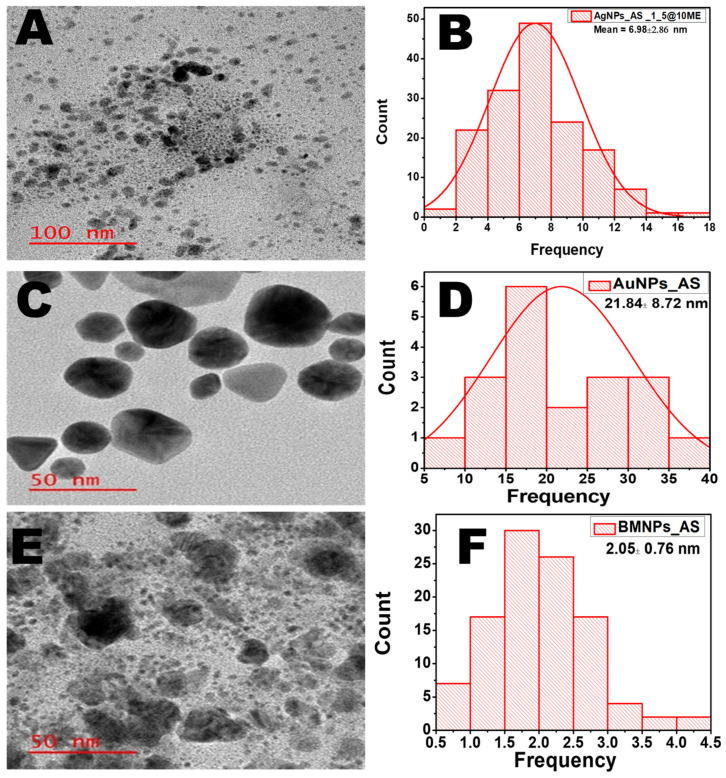
(**A**) TEM micrograph of AgNPs_AS 1:5. (**B**) AgNP particle size distribution. (**C**) TEM micrograph of AuNPs_AS. (**D**) AuNP particle size distribution. (**E**) TEM micrograph of BMNPs. (**F**) BMNP particle size distribution.

The reduction and formation of AgNPs, AuNPs, and BMNPs were first monitored by the color change. The color change from colorless to dark brown was exhibited after adding the aqueous leaf extract of AS to silver nitrate solution (Figure 1(Ai)). The color change in the reaction mixture is indicative of the bio-reduction of Ag ions to AgNPs. At 2 h of reaction, a SPR peak of 444 nm was recorded for the ratio of 1:5 of AS extract/Ag salt, while a SPR peak of 441 nm was recorded for the ratio of 1:10 of AS extract/Ag salt but with a lower absorbance (Figure 1B). The higher absorbance recorded for the 1:5 synthesis was attributed to the more abundant bio-reductant in the reaction mixture. A color change from golden yellow to ruby-red was observed after the bio-reduction of the Au ions to AuNPs upon the addition of the aqueous extract of AS (Figure 1(Aii)). This observed change in color is attributed to the reduction of Au^3+^ to Au^0^. The absorption spectrum shows an SPR peak at 539 nm (Figure 1C), a typical plasmon peak of AuNPs. The bio-reduction of both the Au and Ag salts in a reaction mixture by the AS extract gave a brownish color, as shown in Figure 1(Aiii). The SPR peak of the Ag-Au BMNPs was recorded at 453 nm (Figure 1D). The presence of a single band, as shown in Figure 2C, confirmed the formation of BMNPs. Furthermore, the recorded SPR peak at 453 nm (Figure 1D), a red shift from the plasmon peak of AgNPs but a blue shift from the plasmon peak for AuNPs, further confirmed the formation of the BMNPs and can be considered as a nano-alloy [33]. Furthermore, the closeness of the BMNPs’ SPR peak to that of the AgNPs shows that there was more reduced Ag than Au during the synthesis of the BMNPs. In addition, the observed single SPR peak at the end of the 2 h reaction suggests the completion of the reaction that resulted in the formation of a nano-alloy [34]. Further characterization and application of the AgNPs were performed with the one synthesized using the ratio of 1:5 of AS extract/Ag salt. 

### 2.2. TEM Micrograph of AgNPs, AuNPs, and BMNPs

The TEM micrographs and size distribution of the as-synthesized MNPs are shown in Figure 2. The bio-reduction of the Ag salt by the AS extract for 2 h resulted in small, spherical, and monodispersed AgNPs with an average particle size of 6.98 nm ± 2.86 nm (Figure 2A,B). The AS extract proved to be an efficient bio-reductant that produced uniformly distributed nanoparticles. The synthesis of AuNPs was carried out at room temperature. After 20 h of stirring, small, spherical, and poly-dispersed AuNPs were obtained with an average particle size of 21.84 ± 8.72 nm. Although the size of the AuNPs was larger than that of the AgNPs, the AS extract proved to be an efficient bio-reductant in the reduction of the Au salt without the aid of an external source like heat or a catalyst (Figure 2C,D). On the other hand, the as-synthesized BMNPs_AS image shows monodispersed, spherical, and small NPs with an average particle size of 2.05 nm ± 0.76 nm (Figure 2E,F). The small-sized BMNPs could be attributed to the combined optical, chemical, and electronic properties of the two metals as a result of their synergistic effect [35].

### 2.3. FTIR of AgNPs, AuNPs, and BMNPs

**Figure 3:**
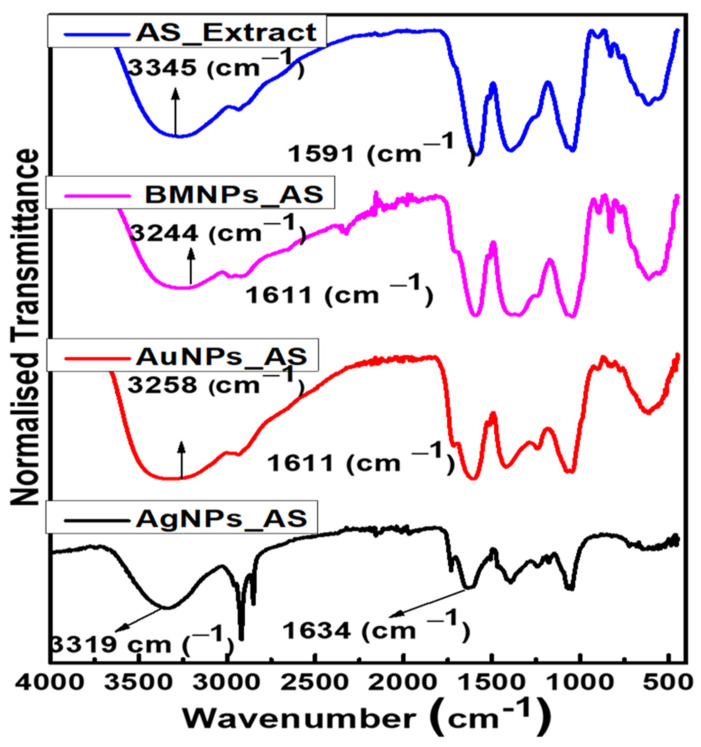
FTIR spectra of the AS extract and the as-synthesized MNPs using the aqueous extract of AS.

The result of the FTIR analysis is shown in Figure 3. The AS extract spectra significantly revealed the functional groups of hydroxyls (–OH) (3345 cm^−1^) and carbonyls (−C=O) (1591 cm^−1^). The as-synthesized AgNPs using AS extract as the bio-reductant showed significant absorption bands at 3319 cm^−1^ and 1634 cm^−1^. These absorption bands correspond to the hydroxyl group of phenols and the symmetric stretch of carbonyl groups, respectively, confirming the presence of ketones and carboxylic acids and thus the role of the AS extract in reducing and stabilizing the AgNPs. Similarly, hydroxyl and carbonyl groups were seen in the as-synthesized AuNPs and BMNPs, as shown in Figure 3. Furthermore, new absorption bands were observed in all the MNPs after the reduction of the metal salt by the AS extract. The observed characteristic peaks of the aqueous AS extract in the AgNPs, AuNPs, and BMNPs show the active role of the AS extract in the efficient reduction and stabilization of the MNPs. The solubility of the MNPs can be ascribed to the presence of the hydroxyl (–OH) group.

### 2.4. XRD of the AgNPs, AuNPs, and BMNPs

**Figure 4:**
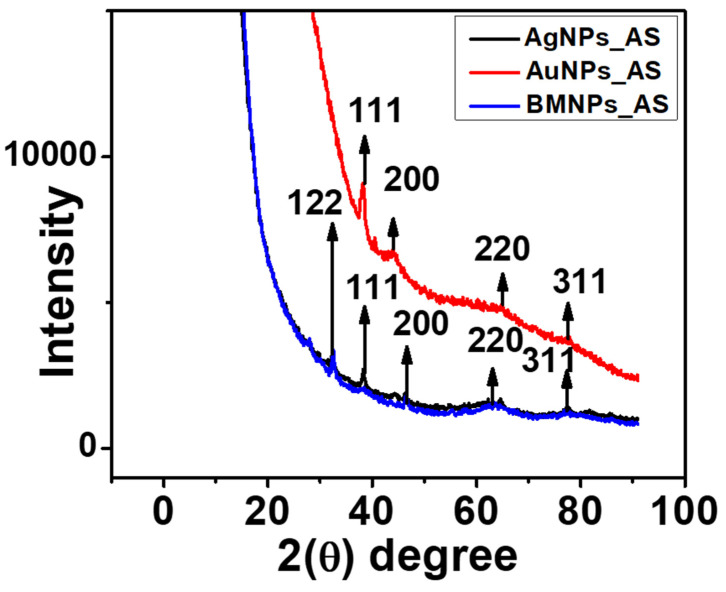
XRD pattern of AgNPs, AuNPs, and BMNPs synthesized using aqueous extract of AS.

The XRD patterns of the as-synthesized MNPs are seen in Figure 4. The XRD confirms the phase and crystallinity of the metal nanoparticles [36]. Five diffraction peaks at 32.83°, 38.10°, 48.86°, 63.72°, and 77.56° were observed for the AgNPs and the BMNPs. These peaks can be assigned to the lattice planes of (122), (111), (200), (220), and (311), relating to the face-centered cubic (FCC) of Ag and correlated to the JCPDS card no: 04-0783 [37]. On the other hand, four diffraction peaks at 37.80°, 40.39°, 64.85°, and 78.19° were seen for the AuNPs (Figure 4), which agree with the face-centered cubic of Au relating to the lattice planes (111), (200), (220), and (311) correlated to the JCPDS card no: 00-004-0784 [38,39]. The observed similarity of the diffraction peak of the BMNPs to that of the AgNPs is a result of the higher composition of the AgNPs in the bimetallic NPs, as shown in the UV absorption spectra in Figure 1D.

### 2.5. Zeta Potential Analysis of AgNPs, AuNPs, and BMNPs

Table 1 shows the DLS analysis of the as-synthesized AgNPs, AuNPs, and BMNPs using the aqueous extract of AS. When two nanoparticles approach one another in a liquid phase, they produce repulsive (electrostatic interaction) and attraction (van der Waals interaction) forces that grow at various scales with shrinking particle distance [40,41]. This causes a peak in the potential energy at close particle distances, which is what gives colloids their stability [42]. The zeta potential of nanoparticle dispersions was calculated using the velocity of the particles under an external electric field [43]. The zeta potential of the as-synthesized AgNPs, AuNPs, and BMNPs was −13.4 mV, +19 mV and −8.71 mV, respectively. The reduced negative charge of the BMNPs results from the positive charge of the AuNPs. The zeta potential results further validate the nanoparticles’ stability in aqueous solutions [44]. The observed PDI values observed for the AgNPs, AuNPs, and BMNPs were 0.485, 0.2814, and 0.2054, which further confirms the uniform distribution of the MNPs. 

### 2.6. Antibacterial Activity of the As-Synthesized MNPs

The microdilution method was applied to assess the antibacterial activity of the as-synthesized MNPs. The concentration range of the MNPs, for which they were tested for antibacterial activities, was from 2000 µg/mL to 1.953 µg/mL. AgNPs_AS and BMNPs_AS both exhibited an inhibitory effect on *S. aureus* and *E. coli*, while the AuNPs_AS had no inhibitory effect on the tested pathogenic organism. AgNPs_AS exhibited an inhibitory effect against *S. aureus* and *E. coli* at a minimum inhibitory concentration value of 62.5 µg/mL and 7.812 µg/mL, respectively. On the other hand, BMNPs_AS showed an inhibitory effect against *S. aureus* and *E. coli* at a much lower MIC of 15.625 µg/mL and 1.953 µg/mL (Table 2). The higher MIC values observed with E. *coli* could be due to their extra lipopolysaccharide on the cell wall of Gram-negative bacteria. 

The very low MIC value observed for the BMNPs_AS against the tested pathogenic bacteria can be attributed to their extremely small size which allowed for the effective permeation and concentration of the BMNPs in the cell wall of the bacteria. Studies have shown that the size of the NPs impacts their efficacy such that smaller-sized NPs are known for better antibacterial activity [45,46]. This is due to the inherent ability of the smaller NPs to penetrate the membranes of the bacterial cells faster due to a larger surface area for penetration. Metal ions are liberated into the cells by oxidation and thus produce ROS that attack the bacterial cells and ultimately lead to cell death [11,12,47,48]. The AuNPs_AS showed no inhibitory effect against the bacterial strain. This might be due to biological inertness based on the inertness of the surface ligands [49].

Also, the shape, antibacterial effects, and size of the synthesized NPs could be linked to the concentration and kind of phytochemicals present in the medicinal plants, and the temperature and reaction time used in their synthesis [50].

## 3. Materials and Methods

### 3.1. Materials

Analytical-grade silver nitrate, gold chloroauric acid, and Mueller–Hinton agar were acquired from Sigma-Aldrich, Johannesburg, South Africa, and Streptomycin (1 mg/mL) was acquired from Sigma-Aldrich, Switzerland (BCBP5897V). Deionized water (DI) was utilized in preparing all the aqueous solutions.

### 3.2. Collection, Authentication, and Preparation of the Plant

The AS plant was procured from the Botanical Garden of the University of Ibadan (UI), Nigeria. The plant was authenticated and deposited at the UI Herbarium with voucher no. UIH-23172. The fully matured, fresh leaves of *A. squamosa* L. (AS) were thoroughly washed using tap water and thereafter with sterilized DI before they were air-dried for some days, and then a blender was used to blend them into a fine powder which was stored in an air-tight jar.

### 3.3. Plant Extraction 

In total, 5 g of AS leaves was added into 100 mL of DI water. The resulting mixture was heated to 60 °C using a sonicator for 10 min. After filtering the mixture, the filtrate was used in the synthesis. 

### 3.4. Biological Synthesis of AgNPs

In total, 6 mL each of the AS extract was added to 30 mL and 60 mL of 10 mM AgNO_3_ in different beakers, respectively (1:5; 1:10). The resulting reaction mixtures were heated at 60 °C for 2 h in a sonicator.

### 3.5. Biological Synthesis of AuNPs

AS extract (5 mL) was added to 25 mL of 1 mM Au chloroauric acid and left to stir at room temperature on a magnetic stirrer at 350 rpm for 20 h. 

### 3.6. Biological Synthesis of BMNPs

AS leaf extract (20 mL) was added to 60 mL of Ag and Au salt (30 mL of 10 mM Ag salt and 30 mL of 1 mM of Au salt) and sonicated at 60 °C for 2 h.

### 3.7. Characterization of the As-Synthesized AgNPs, AuNPs, and BMNPs

In the 200–900 nm range, the absorption spectra were captured with a Perkin Elmer Lambda 25 UV–Vis spectrophotometer (PerkinElmer SA (Pty) Ltd, Midrand, South Africa). Using an acceleration voltage of 200 kV, a JEOL JEM-2100 high-resolution transmission electron microscope (JEOL Ltd, Tokyo, Japan). was employed to determine the morphology of the as-synthesized AgNPs, AuNPs, and BMNPs. To investigate the surface chemistry of the material, FTIR spectroscopy in the 4000–400 cm^−1^ region was performed using a Perkin Elmer Spectrum 2 (PerkinElmer SA (Pty) Ltd, Midrand, South Africa). Using a Bruker D8 Advance X-ray diffractometer (Bruker, Karlsruhe, Germany) and monochromatic Cu-Kα1 radiation (λ = 1.54 Å) at diffraction angles ranging from 10° to 90°, X-ray diffraction (XRD) studies were conducted. The zeta potential of the MNPs was examined using a Malvern Panalytical Zetasizer Nano ZS (Malvern Panalytical Ltd, Malvern, United Kingdom) at 25 °C using the Smoluchowski model.

### 3.8. Antibacterial Activity of the Metallic Nanoparticles (MNPs)

Using the microdilution method, the minimum inhibitory concentration (MIC) of the MNPs was determined according to a previously described method by Fanoro et al. (2021) [50]. Supplementary Material S1 has a detailed description of the process.

## 4. Conclusions

Metallic nanoparticles possess great potential for use as antibacterial agents. In this study, small, spherical, and water-soluble AgNPs, AuNPs, and BMNPs were synthesized through a green facile technique using *Annona squamosa* L. aqueous extract as a bio-reductant and stabilizing agent. The as-synthesized MNPs were adequately characterized by UV, TEM, XRD, FTIR, and DLS to ascertain their size, shape, and other morphological and optical properties that can impact functionality. UV–VIS spectroscopy confirmed the successful synthesis of the MNPs, while FTIR confirmed the role of the AS plant in the reduction and stabilization of the MNPs. The TEM micrographs showed that the average particle size of the as-synthesized nanoparticles was 6.98 ± 2.86 nm, 21.84 ± 8.72, and 2.05 nm ± 0.76 nm for AgNPs_AS, AuNPs_AS, and BMNPs_AS, respectively. BMNPs_AS possessed a much smaller size, which could be ascribed to the synergistic effect between the silver and gold salt. The effect of the small-sized BMNPs_AS consequently resulted in an effective inhibitory role of the as-synthesized BMNPs_AS against *S. aureus,* with a MIC value of 1.953 µg/mL. This work shows that metal NPs synthesized from *Annona Squamosa* L. leaf extract can provide an alternative and indigenous solution for prospecting antibiotics with great potential to fight antibiotic resistance in clinical medicine. Furthermore, we believe that other parts of this plant should be used in the synthesis of nanoparticles in order to explore their medicinal values, which can lead to the development of viable antibiotics. 

## Figures and Tables

**Table 1:** Zeta potential of the as-synthesized MNPs.

S/N	Sample	Zeta Potential (mV)
1	AgNPs_AS	−13.4
2	AuNPs_AS	+19.0
3	BMNPs_AS	−8.71

**Table 2:** MIC results of the antibacterial activity of the MNPs.

S/N	MNPs	*E. coli* (ATCC 25922) (µg/mL)	*S. aureus* (ATCC 25923) (µg/mL)
1.	AgNPs	62.5	7.8125
2.	AuNPs	-	-
3.	BMNPs	15.625	1.953
4.	Streptomycin	1.953	1.953

## Data Availability

The original contributions presented in the study are included in the article/Supplementary Materials, and further inquiries can be directed to the corresponding author.

## Supplementary Materials

The following supporting information can be downloaded at: https://www.mdpi.com/article/10.3390/antibiotics13121199/s1, S1: Antibacterial Activity of the Metallic Nanoparticles (MNPs).
