# Supplementary material for: Biosynthesis, Characterization, and Antibacterial Activity of Gold, Silver, and Bimetallic Nanoparticles Using Annona squamosa L. Leaves
Source: Antibiotics (Basel). 2024 Dec 9;13(12):1199. doi: 10.3390/antibiotics13121199 (PMC11672701; doi:10.3390/antibiotics13121199)
Supplement: Supplementary file 1 [file antibiotics-13-01199-s001.zip › antibiotics-3318196-supplementary.pdf]

Supplementary Data for the Manuscript "Biosynthesis, characterization, and antibacterial activity of gold, silver, and bimetallic nanoparticles using *Annona squamosa* L. leaves".

S.1 Antibacterial Activity of the Metallic Nanoparticles (MNPs)

Fresh bacterial growths of 18 h culture strains of pathogenic bacteria *Staphylococcus aureus* (ATCC 25923) and *Escherichia coli* (ATCC 25922), were standardized to the 0.5 McFarland standards in Muller – Hilton broth and were then used as inoculum. In a 96-well plate containing 100 µL of 1.953, 3.906,7.8125 15.625, 31.25,62.5, 125, 250, 500, 1000, and 2000 µg/mL of each MNPs, 100 µL of each inoculum were seeded in triplicates. The plates were grown overnight at 37 ºC. Muller – Hilton broth (50% *v/v* in DMSO) was utilized as a negative control. Positive control was achieved using Streptomycin (1 mg/mL). Viable cells were confirmed after 2 h incubation, in the presence of resazurin dye, as they enzymatically reduced resazurin dye (blue colour) to the resorufin that fluoresces pink. Bacterial growth was indicated by a pink colour. The lowest concentration that inhibited bacterial growth was recorded as the minimum inhibitory concentration (MIC), and the values were recorded for each bacteria strain.
